# Tunable coupling in magnetic thin film heterostructures with a magnetic phase transition

**DOI:** 10.1038/s41598-023-34322-5

**Published:** 2023-06-12

**Authors:** Kristen Stojak Repa, Brian J. Kirby, Casey W. Miller

**Affiliations:** 1 School of Chemistry and Materials Science, Rochester Institute of Technology, Rochester, NY 14623 USA; 2 Department of Physics, SUNY Brockport, Brockport, NY 14420 USA; 3 NIST Center for Neutron Research, NIST, Gaithersburg, MD 20899 USA

**Keywords:** Condensed-matter physics, Magnetic properties and materials, Surfaces, interfaces and thin films

## Abstract

The magnetic properties of permalloy-based trilayers of the form Py_0.8_Cu_0.2_/Py_0.4_Cu_0.6_/Py/IrMn were studied as the spacer layer undergoes a paramagnetic to ferromagnetic phase transition. We find the coupling between the free Py_0.8_Cu_0.2_ layer and the exchange bias pinned Py to be strongly temperature-dependent: there is negligible coupling above the Curie temperature of the Py_0.4_Cu_0.6_ spacer layer, strong ferromagnetic coupling below that temperature, and a tunable coupling between these extremes. Polarized neutron reflectometry was used to measure the depth profile of the magnetic order in the system, allowing us to correlate the order parameter with the coupling strength. The thickness dependence shows that these are interface effects with an inverse relationship to thickness, and that there is a magnetic proximity effect that enhances the Curie temperature of the spacer layer with characteristic length scale of about 7 nm. As a demonstration of potential functionality of such a system, the structure is shown to spontaneously flip from the antiparallel to parallel magnetic configuration once the spacer layer has developed long-range magnetic order.

## Introduction

Magnetic heterostructures offer a wide variety of opportunities for engineering materials or devices with novel functionalities. Examples include giant magnetoresistance^1^, spin-torque oscillators^2^, and exchange graded films^3^, among others. Such spintronics devices are typically operated at temperatures low relative to the ordering temperature of the magnetic materials of which they are comprised. Heat assisted magnetic recording is an exception in which a dynamic temperature increase, typically caused by a laser pulse, is used to alter the magnetic properties in order to achieve low field switching of high coercivity ferromagnets). Thermomagnetic switching has also been investigated in magnetic heterostructures in relative static environments by sweeping the sample temperature above and below the Curie temperature (*T*_*C*_) of one of the ferromagnetic layers.

Kravets et al.^4,5^ showed that the interlayer coupling in F1/Ni_1−x_Cu_x_/F1 structures could be tuned by changing the temperature above and below the *T*_*C*_ of the nickel-copper alloy. Given that the *T*_*C*_ depends on *x*, a wide temperature range of functionality was demonstrated.

Poulshkin et al.^6^ used the elemental specificity of resonant x-ray magnetic reflectometry of CoFe/NiCu/NiFe heterostructures to show that the magnitude of the interfacial moment was related to the magnetization of the adjacent ferromagnetic layer, and thus larger when contacting CoFe than NiFe. They also showed that the exchange coupling across the interface was sufficiently strong that the interfacial moments of the spacer layer tracked the reversal behavior of the adjacent ferromagnetic layer. Given that the CoFe and NiFe had sufficiently different coercive fields, this imposes upon the NiCu a frustration when the CoFe and NiFe magnetizations are not aligned, which could lead to a spin spiral if the spacer were magnetically ordered.

Intrinsic to these studies is a ferromagnetic proximity effect that exists at the interface between the high *T*_*C*_ materials and the lower *T*_*C*_ spacer layer. Magnetic proximity effects have been studied extensively. For example, Hase et al. used x-ray resonant magnetic scattering to show that for Fe/Pd heterostructures, the Fe layer can induce a large moment in the paramagnetic Pd layer that extends approximately 2 nm from the interface^7^. Engel et al. observed a similar enhancement in Co/Pd superlattices^8^. Other groups have looked at proximity effects with a material whose composition is close to that which would make the alloy ferromagnetic, e.g., Fe/Fe_0.32_V_0.68_ superlattices^9^. In such cases, the proximity effect has been shown to boost the ordering temperature of the alloy well above the alloy’s intrinsic *T*_*C*_^9,10^.

An observation that appears unique to heterostructures that have a low *T*_*C*_ ferromagnet as the spacer is the surprisingly long range of the proximity effect. Whereas proximity effects in systems with paramagnetic spacers are often just a few unit cells^7^, Magnus et al.^11^ estimated at least 30 unit cells remained ferromagnetic above the *T*_*C*_ of Co_60_(AlZ)_40_ in a Co_85_(AlZ)_15_/Co_60_(AlZ)_40_/Sm_90_Co_10_ heterostructure. They also showed coupling between the two ferromagnetic layers persisted for Co_60_(AlZ)_40_ thicknesses of at least 40 nm. Magnetic depth profiles of these samples obtained by polarized neutron reflectometry confirmed this proximity effect.

Here, we study exchange biased spin valve-like structures with a spacer layer that undergoes a ferromagnetic phase transition. This allows for thermal control of ferromagnetic exchange coupling throughout the device. Our structures are comprised of permalloy, a nickel–iron alloy typically consisting of approximately 80% nickel and 20% iron, and permalloy-copper alloys. The *T*_*C*_ of Py_x_Cu_1−x_ decreases linearly by approximately 11 K per atomic-% of Cu from the *T*_*C*_ of pure Py (around 900 K) until the dilution pushes the system into a superparamagnetic regime, theoretically at *x* = 0.83 in the FCC system^12^. The tunability of the system makes it attractive for thermomagnetic applications. Our approach combines traditional magnetometry, which is sensitive to a sample’s total magnetic moment, with polarized neutron reflectometry, which provides depth resolution of magnetic induction within a sample. Using a variety of temperature and field dependent measurements, we find that the strength of exchange bias and related layer coupling is directly correlated with the order parameter of the spacer layer and that this system has a long-range interfacial proximity effect. We also demonstrate the ability of the structure to spontaneously change its magnetic configuration from the antiparallel to parallel state upon cooling.

## Fabrication and methods

We investigated devices of the form SiOx/Py_0.8_Cu_0.2_/Py_0.4_Cu_0.6_/Py/IrMn/Ta, where Py is permalloy (Ni_80_Fe_20_), and Py_x_Cu_1−x_ is permalloy-copper (Py-Cu) alloy. The stoichiometry indicated is that of individual sputtering targets. Samples were grown on thermally oxidized silicon substrates via magnetron sputtering (AJA International ATC Orion-8)^13^ with a base pressure of 8 nTorr (1 Torr = 133 Pa) in an argon atmosphere with a sputtering pressure of 3 mTorr at ambient temperature. The Py and Py-Cu alloy layers were grown with RF sputtering with rates in the range 0.2–0.4 Å/s, depending on the target; IrMn and Ta were grown at 1.2 Å/s and 0.8 Å/s, respectively, using 100 W DC. All samples were grown in the presence of a small magnetic field on the substrate plate to induce exchange bias^14^. The Ta was used to prevent oxidation^15^. Growing directly on oxidized silicon promotes (111) textured growth in the FCC metals, such as Py, Cu, and their alloys^16^. The substrates were rotated continuously during deposition at about 0.5 Hz.

**Figure 1:**
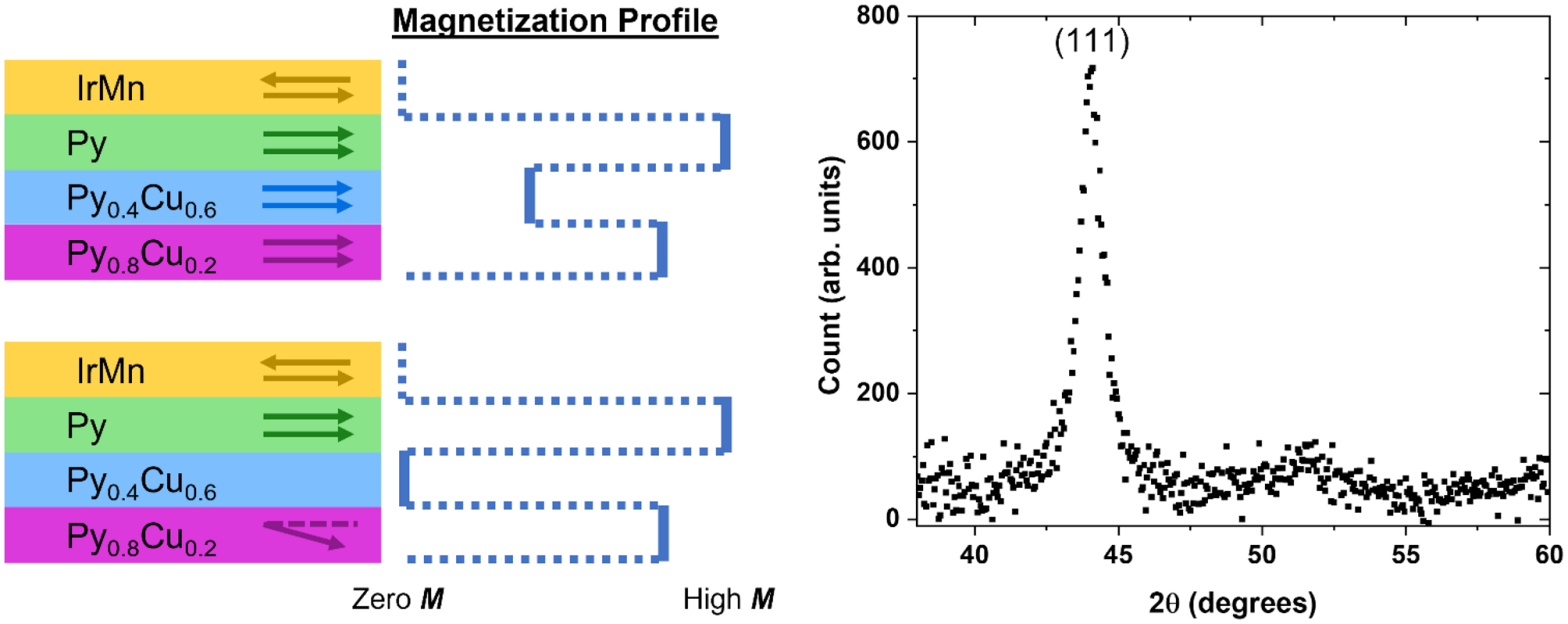
Cartoon of the heterostructures (left) and magnetization profile (middle) at low (top) and high (bottom) temperatures, as described in the text along with x-ray diffraction results (right) showing a peak at (111) for Permalloy.

Figure 1 shows cartoons of the device and the corresponding magnetization profile above and below *T*_*C*_ of the spacer layer along with x-ray diffraction data of Py_0.8_Cu_0.2_ (14 nm)/Py_0.4_Cu_0.6_ (19 nm)/Py (14 nm)/IrMn (7 nm). From top to bottom, the samples are comprised of an antiferromagnetic IrMn layer that pins via exchange bias the magnetization direction of an adjacent high *T*_*C*_ ferromagnetic Py layer, followed by a low *T*_*C*_ ferromagnetic Py_0.4_Cu_0.6_ spacer layer, and a high *T*_*C*_ ferromagnetic Py_0.8_Cu_0.2_ layer. The properties of this synthetic ferromagnet heterostructure are temperature-dependent: above the *T*_*C*_ of Py_0.4_Cu_0.6_ (around 160 K for 20 nm thickness), the spacer is paramagnetic and thus unable to mediate any strong coupling between the adjacent ferromagnetic layers. As such, the multilayer stack behaves as a conventional spin valve at high temperatures, with a Py ‘pinned layer’ and a Py_0.8_Cu_0.2_ ‘free layer.’ When *T* falls below *T*_*C*_ of the spacer, that layer becomes ferromagnetically ordered, which allows ferromagnetic coupling throughout the three ferromagnetic layers. Thus, at low enough temperature, the stack essentially behaves like a single exchange-biased ferromagnet. X-ray diffraction data shows the (111) peak for Py at 2*θ* = 44°, as expected^17^.

DC magnetic measurements were taken using a Quantum Design VersaLab system with the vibrating sample magnetometer option. Magnetization vs. externally applied magnetic field (*M*(*H*)) curves were taken every 10 K between 50 K and 350 K to study temperature dependencies. The applied magnetic field (*H*) was swept from 30 kOe → -30 kOe → 30 kOe (10 kOe = 1 T μ_0_^−1^). This constitutes a major loop, as 30 kOe is sufficient to saturate the magnetizations of all ferromagnetic layers.

Polarized neutron reflectometry (PNR) measurements were conducted at the NIST Center for Neutron Research using the Polarized Beam Reflectometer. This technique allows for deduction of the depth-dependent scattering length density (*ρ*) depth profiles in thin films and multilayers^18^. Non spin-flip reflectivities (+ + and - -) provide information about the nuclear component of *ρ* (indicative of the sample’s nuclear composition), and a magnetic component of ρ proportional to the in-plane magnetization oriented along the applied field direction. Spin-flip reflectivities (+ - and - +) provide information about the in-plane magnetization component perpendicular to *H*. Using a 0.475 nm wavelength neutron beam, the specular spin-dependent reflectivities were measured as functions of wavevector transfer along the sample growth direction (*Q*). The sample was mounted inside a closed-cycle refrigerator, and an electromagnet was used to apply *H* in the sample plane, perpendicular to both the sample growth direction and the neutron propagation direction; the magnitude of the field was changed for different experiments, as described below. Data were reduced using the *Reductus* online software suite^19^. Data modeling was performed using *Refl1D*^20^. The temperature-dependent reflectivities measured in a saturating field were simultaneously fitted, using a model with temperature-dependent magnetic profiles and temperature-independent nuclear profiles. That nuclear profile was held fixed for modeling of the data (except for the scattering length density of the Ta capping layer, which could appear different due to time-dependent issues, e.g., progressive oxidation). All data were modeled as an incoherent addition of scattering from the multilayer structure and that of a bare thermally oxidized Si substrate, in order to account for a small fraction of bare spots on the sample surface. The temperature-dependent magnetic profiles are modeled in terms of three uniformly magnetized layers. This is a simplification, as proximity effects will certainly result in some temperature-dependent changes to the shape of the magnetic profile—particularly near the nominal *T*_*C*_ of the spacer layer^4,21^. However, more complex models did not consistently improve fits to the data. As such, the layer-dependent magnetizations reported in this work should be interpreted as layer-wide averages.

## Measurements and results

### Temperature dependence

**Figure 2:**
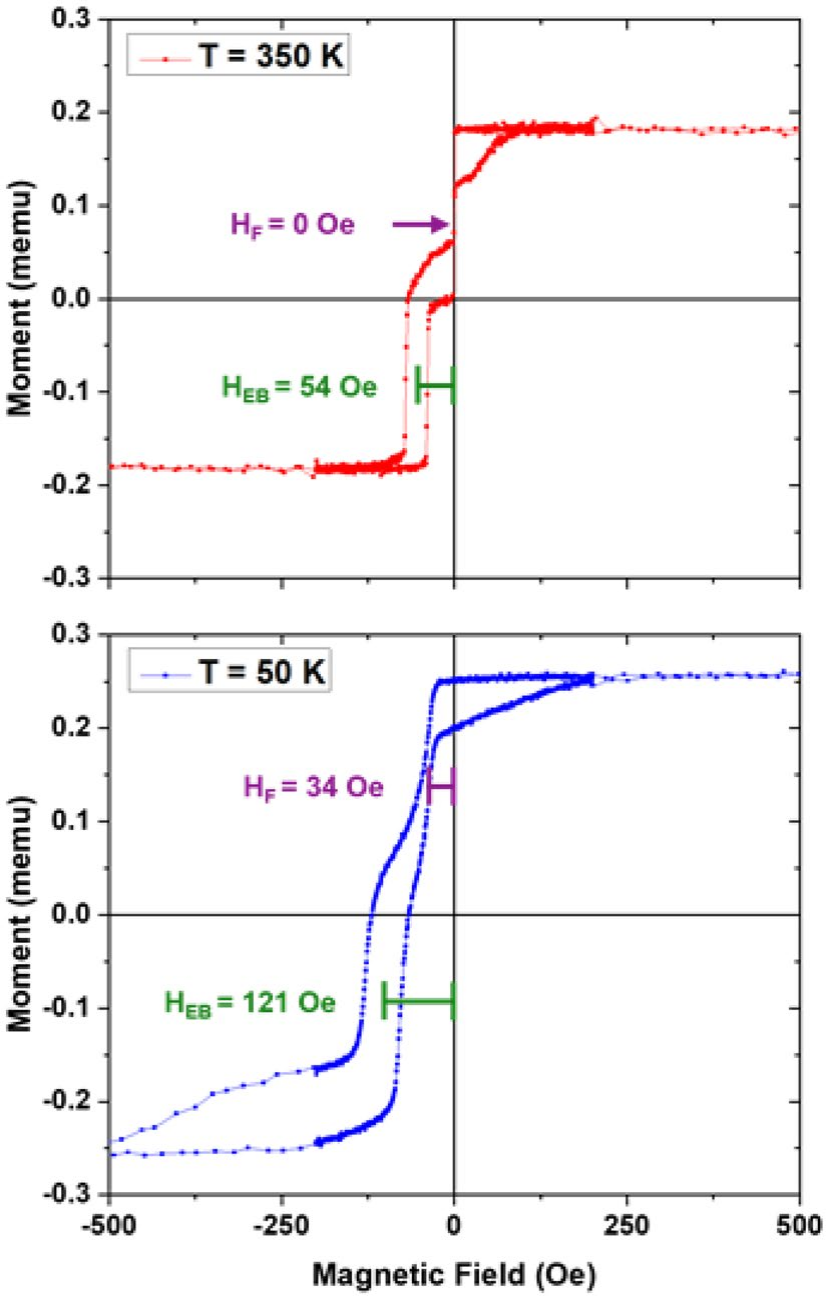
*M*(*H*) curves of Py_0.8_Cu_0.2_ (14 nm)/Py_0.4_Cu_0.6_ (19 nm)/Py (14 nm)/IrMn (7 nm) above *T*_*C*_ (top) and below *T*_*C*_ (bottom) of the spacer with *H*_*EB*_, and *H*_*F*_ shown for both curves.

Figure 2 shows *M*(*H*) for the spin valve above and below *T*_*C*_ of the spacer. We see the hysteresis curve of a typical spin valve above *T*_*C*_. There is a clear magnetic saturation and we can differentiate the switching of the two ferromagnetic layers. The pinned layer (Py) shows exchange bias (*H*_*EB*_ = 54 Oe at 350 K), and has a coercive field of approximately 16 Oe; the free layer (Py_0.8_Cu_0.2_) shows no exchange bias, and has a coercive field of approximately 2 Oe. As we cool the sample below *T*_*C*_ of the spacer, we observe several changes in the *M*(*H*) loops. There is an increase in total moment, consistent with the spacer undergoing a magnetic phase transition. We see *H*_*EB*_ of the pinned layer increase, consistent with expected temperature dependence of an exchange biased bilayer^22^. We also see a bias develop in the free layer when it becomes coupled to the pinned layer. Related phenomena have been reported elsewhere^4,5^. We will refer to the shift of the free layer’s hysteresis loop as the coupling field, *H*_*F*_, and retain *H*_*EB*_ for the pinned layer. This shift, *H*_*F*_, resembles exchange bias, and has a related explanation. The Py layer is positively saturated throughout the reversal of the free layer. The ferromagnetic coupling between the pinned and the free layer mediated by the ferromagnetic spacer layer imposes a torque that opposes[aids] the free layer reversal for negative[positive] sweeping field. This leads the hysteresis loop of the free layer to shift to negative field by an amount *H*_*F*_.

**Figure 3:**
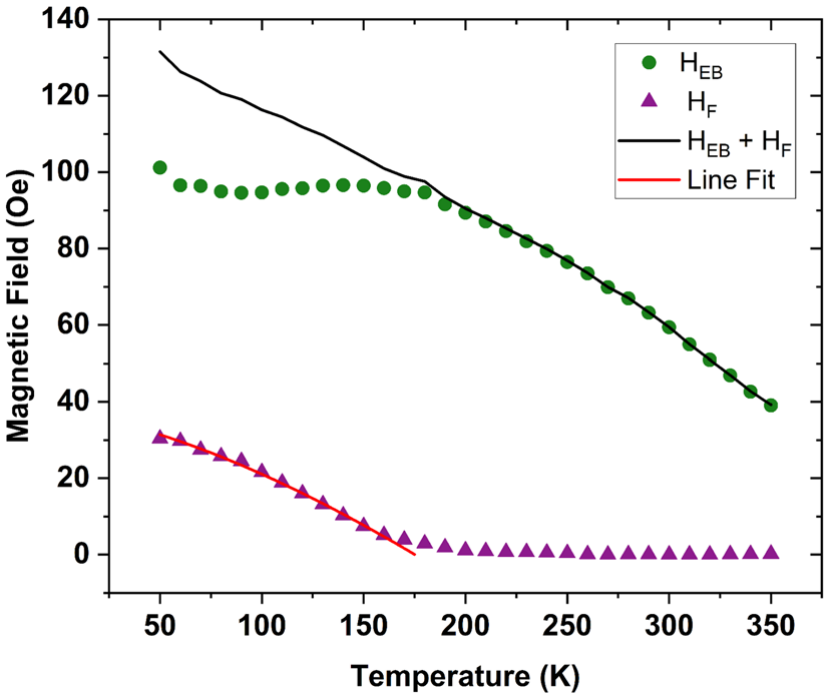
Temperature dependence of the free layer coupling field *H*_*F*_ (purple triangles) and exchange bias field *H*_*EB*_ (green circles). The sum *H *_*F*_ + *H*_*EB*_ (solid line) is the temperature dependence of the IrMn/Py in the absence of any coupling to the free layer. Both curves shown are of Py_0.8_Cu_0.2_ (14 nm)/Py_0.4_Cu_0.6_ (19 nm)/Py (14 nm)/IrMn (7 nm)^23^.

Figure 3 shows the temperature evolution of *H*_*F*_ and *H*_*EB*_. Because the spacer magnetization increases with decreasing temperature, the coupling field, *H*_*F*_, does as well. This behavior is directly analogous to that observed for a typical exchange biased bilayer cooled through the blocking temperature of the antiferromagnet. For *H*_*EB*_, the coupling caused by the spacer’s phase transition leads to a reduction of *H*_*EB*_ for temperatures below the spacer's *T*_*c*_, leading to a deviation from a typical exchange bias temperature dependence. Interestingly, the sum of these fields, *H*_*F*_ + *H*_*EB*_, shows the smooth temperature dependence expected in exchange biased systems. This is explainable by considering the torques imposed on the pinned Py layer from the IrMn at one interface and the Py_0.4_Cu_0.6_ layer at the other interface. The coupling to the antiferromagnet causes the Py hysteresis loop to shift to negative fields by opposing[assisting] the reversal of the magnetization for the negative[positive] sweeping field. Since the Py_0.4_Cu_0.6_ will couple ferromagnetically to the Py and is negatively saturated throughout the duration of the Py layer switching, it assists[opposes] the Py layer switching for the negative[positive] field sweeping direction. Thus, the torques acting upon the Py layer by the IrMn and Py_0.4_Cu_0.6_ oppose each other once the Py_0.4_Cu_0.6_ has long range order. This leads to a reduction of the magnitude of *H*_*EB*_ from that of the isolated IrMn/Py behavior. We can then recover the intrinsic temperature dependence by adding the measured *H*_*F*_ to the measured *H*_*EB*_.

**Figure 4:**
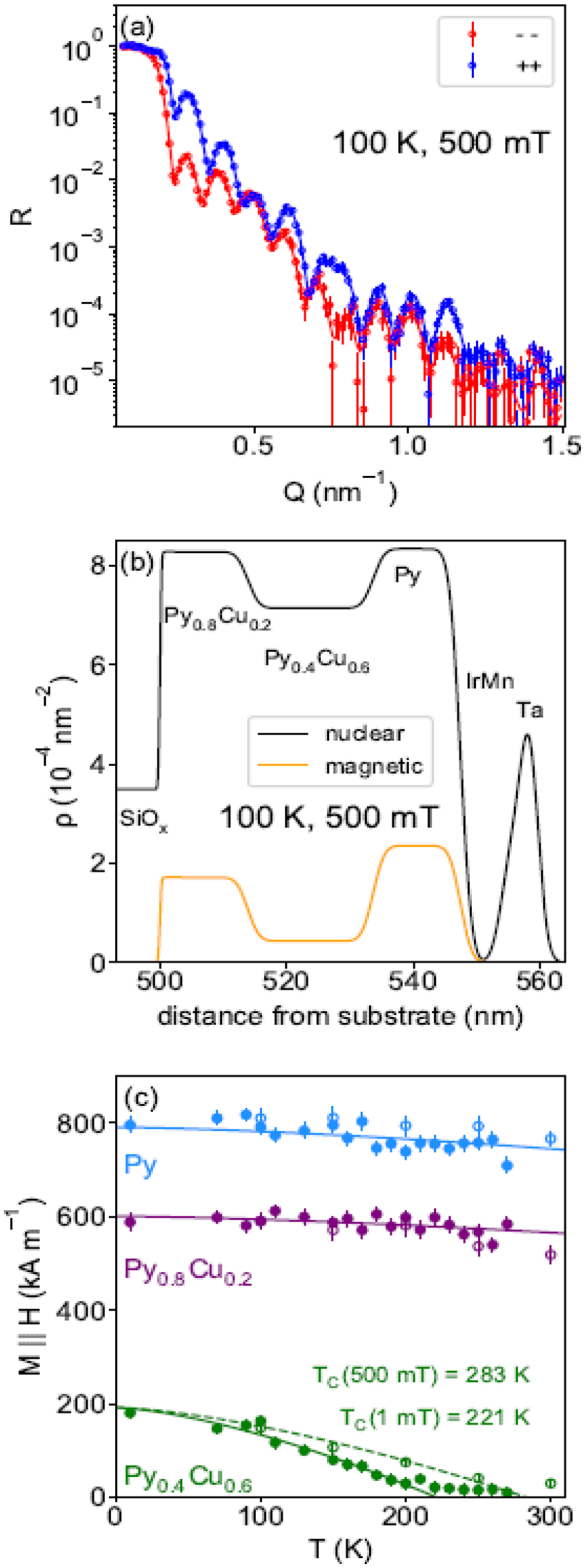
(**a**) Representative fitted PNR data for Py magnetization parallel to *H*. (**b**) Scattering length depth profile used to fit the data in (**a**). The nuclear profile shown was used for all fitting described in this work. (**c**) Temperature-dependent layer-by-layer magnetizations for remenant (closed symbols) and saturation (open symbols) field conditions. Note: 1 mTμ_0_^−1^ = 10 Oe. All curves of Py_0.8_Cu_0.2_ (14 nm)/Py_0.4_Cu_0.6_ (19 nm)/Py (14 nm)/IrMn (7 nm).

We also studied the temperature dependence of the structure with PNR to explicitly resolve the layer-dependent magnetization. Temperature-dependent PNR measurements were taken with magnetic fields nominally parallel to the exchange anisotropy axis and with magnitudes of 5 kOe (at saturation) and 10 Oe (at remenance). Only non spin-flip reflectivities were measured for these conditions because we expected no appreciable magnetization component perpendicular to *H* (and thus no spin-flip scattering). Figure 4a shows example fitted data at 100 K and 5 kOe for a sample with a nominal spacer thickness of 19 nm. The model fits closely match the data; the corresponding nuclear and magnetic scattering length density profiles are shown in Fig. 4b. The nuclear and magnetic scattering length densities of Py are larger than that of Cu, thus the increase in both values with Py concentration is consistent with expectations. The thicknesses of the magnetically active outer and spacer layers are 14 nm and 19 nm, respectively, also consistent with expectations. The corresponding saturation and remenant layer-by-layer temperature-dependent magnetizations are shown in Fig. 4c. Bloch-Law fits are shown as lines through the data. The pinned Py and free Py_0.8_Cu_0.2_ layers’ magnetizations are essentially field-independent, as their *T*_*C*_ values are both well above room temperature. The Bloch-Law fits indicate that with a small applied magnetic field of 10 Oe, the spacer layer has approximately *T*_*C*_ = 221 K, with this seemingly enhanced to 283 K under the 5 kOe saturation field. The inflection point (max d*M*/d*T*) is dramatically higher for the 5 kOe saturation field than would normally be expected, hence this is a large temperature difference for such a small field change, compared to phase transitions in bulk ferromagnetic materials^24^.

**Figure 5:**
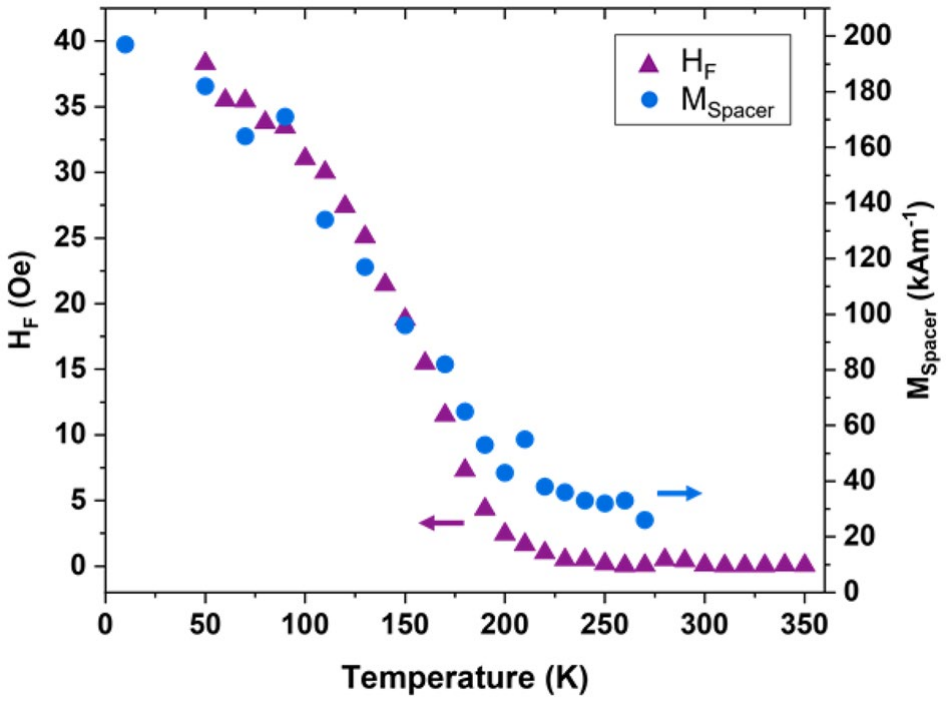
The correlation of the temperature dependences of the coupling field of the free layer (triangles, left axis) from VSM and magnetization of the spacer layer (circles, right axis) from PNR depth profiles show that the spacer layer mediates a tunable coupling between the free and pinned layers of Py_0.8_Cu_0.2_ (14 nm)/Py_0.4_Cu_0.6_ (19 nm)/Py (14 nm)/IrMn (7 nm).

Combining the conventional magnetometry results with those of the PNR gives us some insight into the physics underlying the behavior of these structures. Figure 5 (left axis) shows the temperature dependence of *H*_*F*_, as measured by VSM. Figure 5 (right axis) shows the temperature dependence of the spacer layer’s magnetization, as measured by PNR. The correlation is obvious. Together, these data indicate that the coupling of the free layer to the pinned layer is mediated through the spacer, with the strength of that coupling being dictated by the magnetic order parameter of the spacer layer. The order parameter of the spacer thus serves as a knob that can tune the coupling between the free and pinned layers.

### Thickness dependences

**Figure 6:**
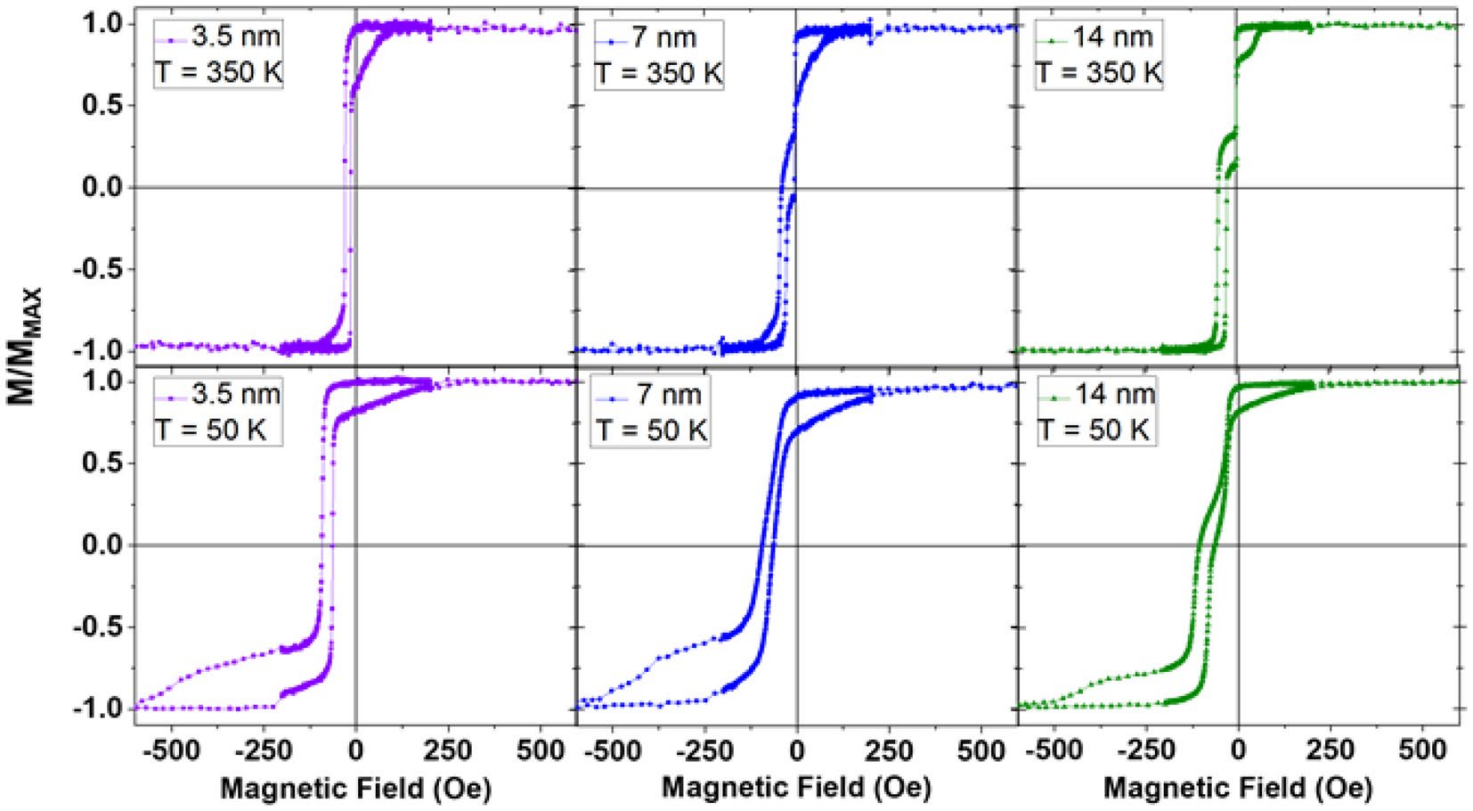
Hysteresis loops above (top row) and below (bottom row) the spacer layer’s *T*_*C*_, for Py_0.8_Cu_0.2_ (14 nm)/Py_0.4_Cu_0.6_ (3.5 nm, 7 nm, and 14 nm)/Py (14 nm)/IrMn (7 nm).

We also investigated the behavior of this system as a function of the spacer layer thickness with samples of the form SiOx/Py_0.8_Cu_0.2_/Py_0.4_Cu_0.6_(t)/Py/IrMn/Ta, where *t* ranged from 3.5 nm to 71 nm with logarithmic spacing. Figure 6 shows example hysteresis loops above and below the Curie temperature for three spacer layer thicknesses. For samples with *t* = 14 nm or more (right, green in Fig. 6), we see behavior similar to that shown in Fig. 2: there is distinct switching of the pinned and free layers at high temperature, with a smearing of the loops when the spacer layer has long range order at low temperatures. The thinnest sample, *t* = 3.5 nm (left, purple in Fig. 6), however, shows the layers to be completely coupled even at high temperatures; no distinct loops can be discerned for the pinned and free layers. This strong coupling may imply samples of this thickness are fundamentally different from the others. The *t* = 7 nm sample (middle, blue in Fig. 6) shows the transition between these two extremes. Other groups^4^ have made similar thermomagnetic devices and conducted interlayer thickness studies, however they found that coupling diminishes beyond an interlayer thickness of approximately 10 nm. In our studies, we determine that (at least partial) coupling exists at interlayer thicknesses of up to approximately 70 nm. We believe that this long range magnetic coupling interaction could happen for many reasons. For example, it could be that this system has highly enhanced susceptibility, which enables the distance between the ferromagnet layers to be artificially enhanced (longer than typically expected). It’s possible that the enhanced susceptibility could be related to some of the layers being diluted ferromagnets. Another possibility is that there could be clusters of Py within the spacer layer, each of which may again help to propagate the magnetic state to longer length scales than if the spacer were pure copper. We also note that exchange coupling becomes too strong for the device to properly function as a spin valve with interlayer thicknesses of 3.5 nm.

From an exchange spring perspective^25^, the stronger *H*_*F*_ for thinner films suggests the spacer is unable to support a domain wall or twist of the magnetization within its thickness. In contrast, the thicker samples may allow such a twist or even full domain wall. That would allow the free layer magnetization to reverse at lower fields, even in the case that the order parameter of the spacer was independent of thickness. This would lead to an inverse relationship between *H*_*F*_ and *t*, ultimately with the thinnest sample showing full coupling within the ferromagnetic layers. The transition between the strong and weak coupling appears to be for a thickness around 7 nm.

**Figure 7:**
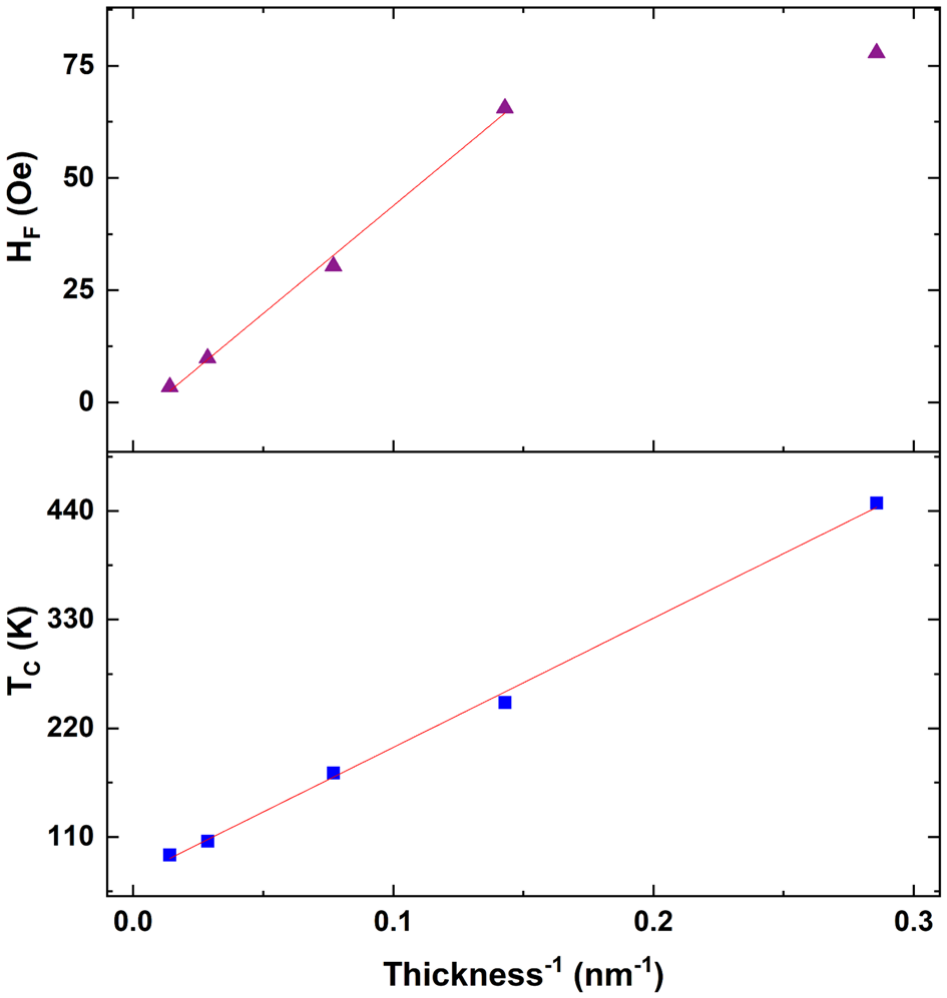
The coupling field (top, purple triangles) and spacer layer Curie Temperature (bottom, blue squares) are inversely related to the spacer layer thickness. The red line is a linear fit. These are Py_0.8_Cu_0.2_ (14 nm)/Py_0.4_Cu_0.6_ (3.5–71 nm)/Py (14 nm)/IrMn (7 nm).

The thickness dependencies of *H*_*F*_ and the spacer layer *T*_*C*_ both scale inversely with thickness, as shown in Figure 7. The coupling deviates from this behavior for the thinnest (3.5 nm) sample; this could be due to roughness/orange-peel coupling becoming dominant for such a thin layer. Fitting the *H*_*F*_(*t*) = *he*^*-t/λ*^ to a decaying exponential for the thicker samples reveals a characteristic length scale of λ = 7.0 ± 1.5 nm. This is a long length scale for a magnetic proximity effect, but consistent with observations in other systems^11,21^. This may have some enhancement because of the structural coherence of the system, in that all the layers are FCC structure with minor differences in lattice parameters. Doing a similar fit of *T*_*C*_(*t*) reveals a length scale of 4.7 nm ± 1.0 nm. To further investigate proximity effects on the spacer’s *T*_*C*_, additional temperature-dependent PNR measurements were taken at 500 Oe to obtain *M* vs.* T* data (similar to Fig. 5) from samples with different spacer thicknesses. Relative to a *T*_*C*_ of 145 K ± 7 K for an isolated 27 nm thick film of the spacer material, we found *T*_*C*_ for *t* = 71 nm and *t* = 13 nm films to be 158 K ± 7 K and 226 K ± 12 K, respectively. Though we only have two data points from PNR at this time, this inverse relationship between spacer thickness and ordering temperature is a clear signature of a magnetic proximity effect^9,11,21,26^, which supports the conclusions drawn from conventional magnetometry data.

## Thermally induced switching

**Figure 8:**
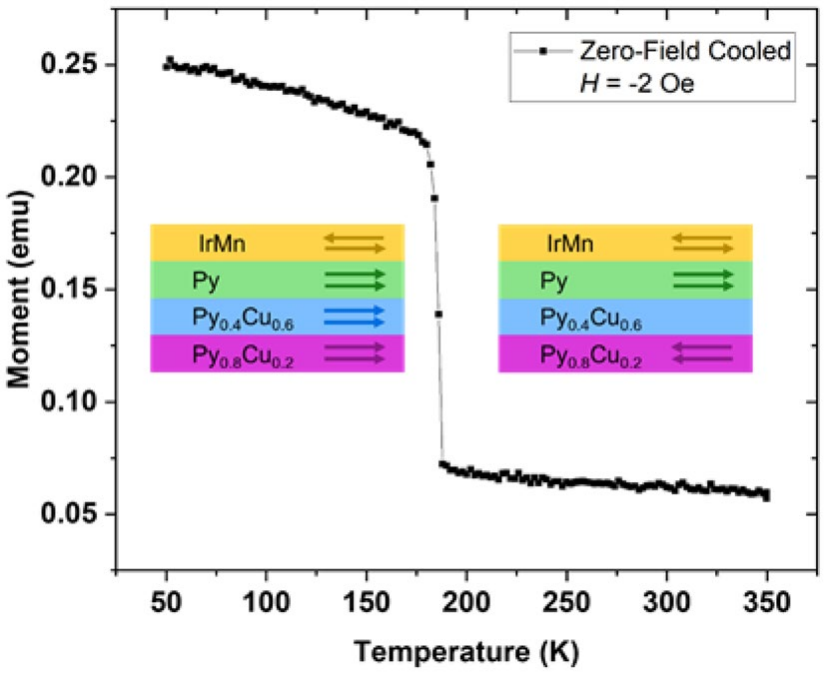
When the device is set to the antiparallel configuration at room temperature, then cooled through the Curie point of the spacer layer, the free layer rotates to align with the pinned layer, resulting in an increase in the net magnetic moment measured by VSM. This is for Py_0.8_Cu_0.2_ (14 nm)/Py_0.4_Cu_0.6_ (19 nm)/Py (14 nm)/IrMn (7 nm).

The onset of coupling between the pinned and free layers at low temperatures can lead to a spontaneous reversal of the free layer. As demonstrated in Figure 8 the free layer will rotate its moment to align with that of the pinned layer when the device is set into the antiparallel state then cooled through the phase transition of the spacer layer. SiOx/Py_0.8_Cu_0.2_(14 nm)/Py_0.4_Cu_0.6_(20 nm)/Py(13 nm)/IrMn(7 nm)/Ta(5 nm). Figure 8 shows the total moment of the sample as a function of temperature, as measured by VSM. The transition is related to the total moment being low in the antiparallel state (the free and pinned magnetizations are pointed in opposite directions and thus effectively cancel each other) and high in the parallel state (free and pinned magnetizations are pointed in the same direction). The strong change of net magnetic moment could be useful for in sensing and possibly energy harvesting.

**Figure 9:**
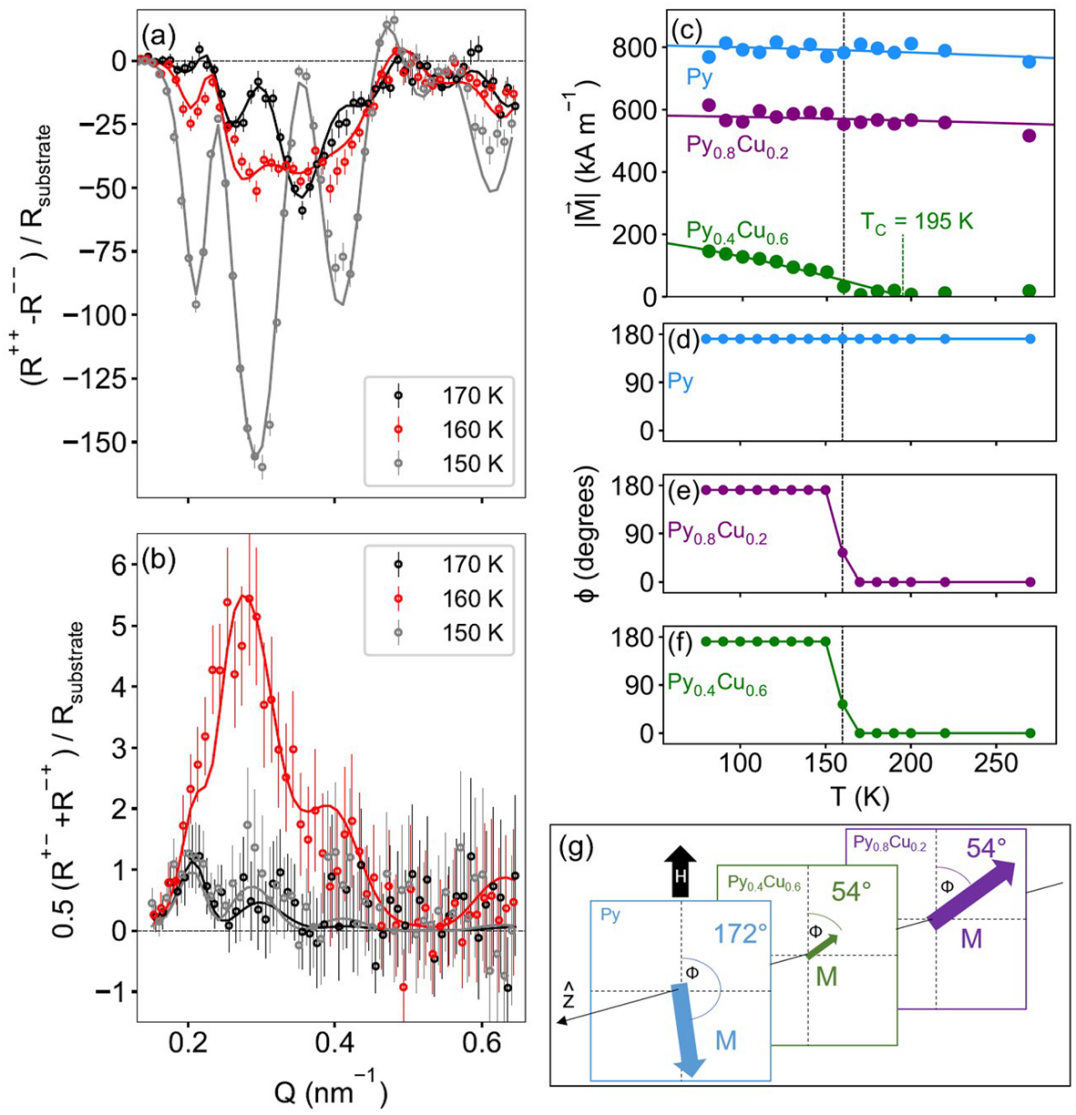
Summary of PNR results with Py magnetization nominally antiparallel to *H* (10 Oe) of Py_0.8_Cu_0.2_ (14 nm)/Py_0.4_Cu_0.6_ (19 nm)/Py (14 nm)/IrMn (7 nm). (**a**) Fitted non spin-flip data near the thermal switching transition temperature. (**b**) Fitted spin-flip data near the transition. (**c**–**f**) Summary of the temperature-dependent magnetization parameters used to fit the data. (**g**) Depiction of the sample during thermal switching at 160 K.

PNR was used to directly characterize the depth-dependence of the thermal switching phenomenon, as shown in Figure 9. At room temperature, well above the nominal *T*_*C*_ of the spacer layer, the sample was oriented with the pinned Py magnetization antiparallel to a 10 Oe applied field used to orient the magnetization of the low anisotropy free layer. Both non spin-flip and spin-flip data were measured as a function of decreasing temperature in order to characterize the layer-dependent magnetization rotation as the spacer layer becomes magnetically ordered and thus capable of mediating direct exchange between the free and pinned layers. Inspection of the data suggest a sharp magnetic switching between 170 and 150 K, as data taken above and below those two temperatures respectively are only weakly temperature-dependent. Fitted non spin-flip data taken near the switching temperature are shown in Fig. 9a, plotted as the difference between +  + and - -, normalized by the theoretical reflectivity of the bare substrate. As the nuclear contribution to +  + and - - is essentially temperature independent, changes in the difference track changes to the component of the in-plane magnetization parallel to *H*. The corresponding fitted spin-flip data are shown in Fig. 9b, plotted as the average of + - and - + (which are identical under the conditions here), again normalized by the substrate reflectivity. These data track changes to the component of the in-plane magnetization perpendicular to *H*. The drastic change in non spin-flip reflectivities and the spike in spin-flip reflectivities at 160 K indicate a switching from nominally antiparallel to nominally parallel alignment of the free and pinned layers. The data are well-fit by a highly constrained model parameterized in terms each layer’s magnetization magnitude, and the angle (*ɸ*) between that magnetization and *H*. Specifically, the model assumes:

- An uncoupled to coupled transition corresponding to the changes in scattering at 160 K;
- *ɸ* for the pinned Py layer is *T*-independent, and is treated as a free parameter to account for misalignment from the desired 180 degree orientation with *H;*
- At *T* = 170 K and above, *ɸ* = 0 degrees for the free and spacer layers;
- At *T* = 160 K, *ɸ* is constrained to be the same for the free and spacer layers, and is treated as a free parameter;
- At *T* = 150 K and below, *ɸ* for all three layers are the same.

The good fits demonstrate that the data are consistent with the above constraints. More complex models, or those with more parameter freedom, do not result in significantly better fits to the data. Most notably, the magnetization of the spacer layer is small enough near the 160 K transition, that the direction cannot be reliably determined as an independent parameter. The modeling results are summarized in Fig. 9 (c-f). At high *T*, the pinned layer (Py) magnetization is pinned at an angle of 8 degrees away from antiparallel with *H*, the spacer layer (Py_0.4_Cu_0.6_) has zero magnetization, and the free layer (Py_0.8_Cu_0.2_) magnetization is aligned parallel with *H*. Below 200 K, the Py_0.4_Cu_0.6_ layer begins to magnetically order, and below 170 K the interlayer coupling mediated via that layer becomes strong enough to reorient the free layer. At 160 K we capture the sample in transition as depicted in Fig. 9g. At 150 K, the interlayer coupling is strong enough to have completely oriented the free and spacer layer magnetizations parallel to that of the pinned Py. Notably, the apparent *T*_*C*_ of the Py_0.4_Cu_0.6_ layer is 195 K in this antiparallel configuration, which is 25 K lower than what was observed for the parallel state to *H* (Fig. 9c). The *T*_*C*_ difference is likely a consequence of dueling proximity effects at the two Py_0.4_Cu_0.6_ interfaces; related phenomena have been seen in superconducting heterostructures^27^. The magnetization profile within the Py_0.4_Cu_0.6_ layers may well even be twisted near the transition temperature, but such detail was not possible to distinguish with our measurements.

## Conclusions

Exchange biased spin-valve-like structures with a spacer layer that undergoes a magnetic phase transition exhibit novel phenomena. We showed that these phenomena are interface effects with an inverse relationship to thickness. The magnetic proximity effect, an enhancement of the ordering temperature of the spacer, has a relatively long length scale of about 7 nm in this system. The coupling between the free and pinned layers is strongly affected by the magnetic order within the spacer layer. With sufficient order, the system is fully ferromagnetically coupled. With no long-range magnetic order, the system is essentially decoupled, allowing the outer layers to switch independently. Between these two extremes, the coupling is strongly dependent on magnetic order and thus temperature. The nominally free layer acquires a bias due to this coupling, which manifests as a shift of its hysteresis loop toward that of the exchange biased pinned layer until they ultimately merge when sufficient long range magnetic order is present in the spacer. This coupling follows a temperature dependence that is strongly correlated with that of the spacer layer’s order parameter. We demonstrated this by showing that the system prepared in the anti-parallel magnetic configuration spontaneously rotates into the parallel configuration when the temperature crosses a threshold related to the establishment of long-range magnetic order in the spacer. This suggests the potential for novel thermo-magnetic devices in which the spacer layer influences the state of the device.

## Data Availability

All data is available. Please e-mail B.J.K for PNR data, and the corresponding author (K.S.R.) for all other data.

## References

[1] Nakamura K, Thomas H (1988). Quantum billiard in a magnetic field: Chaos and diamagnetism. Phys. Rev. Lett..

[2] Awad AA, Dürrenfeld P, Houshang A, Dvornik M, Iacocca E, Dumas RK, Åkerman J (2017). Long -range mutual synchronization of spin Hall nano-oscillators. Nat. Phys..

[3] Kirby B, Belliveau HF, Belyea DD, Kienzle PA, Grutter AJ, Riego P, Berger A, Miller CW (2016). Spatial evolution of the ferromagnetic phase transition in an exchange graded film. Phys. Rev. Lett..

[4] Kravets AF, Timoshevskii A, Yanchitsky BZ, Bergmann MA, Buhler J, Andersson S, Korenivski V (2012). Temperature-controlled interlayer exchange coupling in strong/weak ferromagnetic multilayers: A thermomagnetic Curie switch. Phys. Rev. B.

[5] Kravets AF, Dzhezherya YI, Tovstolytkin AI, Kozak IM, Gryshchuk A, Savina YO, Pashchenko VA, Gnatchenko SL, Koop B, Korenivski V (2014). Synthetic ferrimagnets with thermomagnetic switching. Phys. Rev. B.

[6] Polushkin NI, Kravtsov EA, Vdovichev SN, Tatarskiy DA, Drozdov MN, Weschke E, Fraerman AA (2020). Thermomagnetic switching in strong/weak/strong ferromagnetic, stack detected with resonant x-ray magnetic refelectometry. J. Magn. Magn. Mater..

[7] Hase TPA, Brewer MS, Arnalds UB, Ahlberg M, Kapaklis V, Björck V, Bouchenoire L, Thompson P, Haskel D, Choi Y, Lang J, Sánchez-Hanke C, Hjörvarsson B (2014). Proximity effects on dimensionality and magnetic ordering in Pd/Fe/Pd trilayers. Phys. Rev. B.

[8] Engel BN, England CD, Van Leeuwen R, Nakada M, Falco CM (1991). Magnetic properties of epitaxial Co/Pd superlattices. J. Appl. Phys..

[9] Palonen H, Magnus F, Hjörvarsson B (2018). Double magnetic proximity in Fe/Fe_0.32_V_0.68_ superlattices. Phys. Rev. B.

[10] Ahlberg M, Korelis P, Andersson G, Hjörvarsson B (2012). Effect of ferromagnetic proximity on critical behavior. Phys. Rev. B.

[11] Magnus F, Brooks-Bartlett ME, Moubah R, Procter RA, Andersson G, Hase TPA, Banks ST, Hjörvarsson B (2016). Long-range magnetic interactions and proximity effects in an amorphous exchange-spring magnet. Nat. Commun..

[12] Qader MA, Vishina V, Yu Y, Garcia C, Singh RK, Rizzo ND, Huang M, Chamberlin R, Belashchenko KD, van Schilfgaarde M, Newman N (2017). The magnetic, electrical and structural properties of copper-permalloy alloys. J. Magn. Magn. Mater..

[13] Certain commercial products are identified here to describe our study adequately. Such identification is not intended to imply recommendation or endorsement by the National Institute of Standards and Technology.

[14] Jayathilaka PB, Bauer CA, Williams DV, Miller CW (2010). Influence of growth field on NiFe, Fe_3_O_4_, and NiFe/Cr/Fe_3_O_4_ spin-valves. IEEE Trans. Magn..

[15] Gan L, Gomez RD, Powell CJ, McMichael RD, Chen PJ, Egelhoff WF (2003). Thin Al, Au, Cu, Ni, Fe, and Ta films as oxidation barriers for Co in air. J. Appl. Phys..

[16] Bolon BT, Haugen MA, Abin-Fuentes A, Deneen J, Carter CB, Leighton C (2007). Multiple antiferromagnet/ferromagnet interfaces as a probe of grain-size-dependent exchange bias in polycrystalline Co/Fe_50_Mn_50_. J. Magn. Magn. Mater..

[17] Lee WY, Toney MF, Mauri D (2000). High magnetoresistance in sputtered permalloy thin films through growth on seed layers of (Ni081Fe019)1-xCrx. IEEE Trans. Magn..

[18] Majkrzak, C. F., O’Donovan, K. V., and Berk, N. F. Polarized Neutron Reflectometry in *Neutron Scattering from Magnetic Materials* (ed. Chatterji, T.) 397–471 (Elsevier, 2006).

[19] Maranville B, Ratcliff W, Kienzle P (2018). *Reductus*: A stateless Python data reduction service with a browser front end. J. Appl. Cryst..

[20] Kirby BJ, Kienzle PA, Maranville BB, Berk NF, Krycka J, Heinrich F, Majkrzak CF (2012). Phase-sensitive specular neutron reflectometry for imaging the nanometer scale composition depth profile of thin-film materials. Curr. Opin. Colloid. In..

[21] Qviller AJ, Frommen C, Hauback BC, Magnus F, Kirby BJ, Hjörvarsson B (2020). Direct observation of magnetic proximity effects in amorphous exchange-spring magnets by neutron reflectometry. Phys. Rev. Mater..

[22] Nogués J, Schuller IK (1999). Exchange bias. J. Magn. Magn. Mater..

[23] We believe the open features on the *M(H)* loops to be from background soft magnetic phase, possibly from deposition on the substrate edges; it is present in uniform films measured by VSM, but does not appear in Kerr effect measurements.

[24] Miller CW, Belyea DD, Kirby BJ (2014). Magnetocaloric effect in nanoscale thin films and heterostructures. J. Vac. Sci. Technol. A.

[25] Fullerton EE, Jiang JS, Bader SD (1999). Hard/soft magnetic heterostructures: model exchange-spring magnets. J. Magn. Magn. Mater..

[26] Thórarinsdóttir KA, Palonen H, Palsson GK, Hjörvarsson B, Magnus F (2019). Giant magnetic proximity effect in amorphous layered magnets. Phys. Rev. Mater..

[27] Gu JY, You C-Y, Jiang JS, Pearson J, Bazaliy YB, Bader SD (2002). Magnetization-orientation dependence of the superconducting transition temperature in the ferromagnet-superconductor-ferromagnet system: CuNi/Nb/CuNi. Phys. Rev. Lett..

